# The Anti-Proliferation Activity and Mechanism of Action of K_12_[V_18_O_42_(H_2_O)]∙6H_2_O on Breast Cancer Cell Lines

**DOI:** 10.3390/molecules22091535

**Published:** 2017-09-12

**Authors:** Wen Qi, Boyu Zhang, Yanfei Qi, Shuanli Guo, Rui Tian, Jiaheng Sun, Mingming Zhao

**Affiliations:** School of Public Health, Jilin University, Changchun 130021, Jilin, China; qiwen16@mails.jlu.edu.cn (W.Q.); Zhangboyu_1993@163.com (B.Z.); guosl15@mails.jlu.edu.cn (S.G.); Tianrui16@mails.jlu.edu.cn (R.T.); sunjh15@mails.jlu.edu.cn (J.S.); doubleming1993@163.com (M.Z.)

**Keywords:** polyoxometalates, anti-proliferation, DNA binding effect, BSA binding effect

## Abstract

Polyoxometalates (POMs) are inorganic clusters that possess potential anti-bacterial, anti-viral, and anti-tumor activities. Herein, the in vitro anti-proliferation activities of K_12_[V_18_O_42_(H_2_O)]∙6H_2_O (**V_18_**) have been investigated on the MCF-7 and MDA-MB-231 cell lines. The results indicated that **V_18_** could inhibit the proliferation of MCF-7 (IC_50_, 11.95 μM at 48 h) in a dose-dependent manner compared to the positive control, 5-fluorouracil (5-Fu, *p* < 0.05). The anti-proliferation activity of **V_18_** might be mediated by arrest of the MCF-7 cells in the G_2_/M phase and induction of apoptosis and necrosis. Moreover, **V_18_** can effectively quench the fluorescence of *ct*DNA. The binding mode between them may be groove or outside stacking binding. **V_18_** can also effectively quench the intrinsic fluorescence of bovine serum albumin (BSA) and human serum albumin (HSA) via static quenching, and changed the conformation of BSA and HSA.

## 1. Introduction

Polyoxometalates (POMs) have received wide attention because of their versatile structures and various applications in chemistry, materials science, catalysis, redox, magnetism, and medicine [1,2,3]. In recent years, POMs have been extensively studied in the medical field due to their anti-tumor and anti-bacterial activity [4,5]. Some researchers have reported that POMs can inhibit the growth of breast, colon, stomach, and other cancers [6,7,8]. So far, the anti-tumor mechanism of POMs is not entirely clear. Yamase and co-workers first attributed the inhibitory effect against cancers to a single electron reduction/oxidation cycle in human MX-1 breast cancer cells, OAT lung cancer cells, and Co-4 colon cancer cells [9,10]. Moreover, it has been found that POMs could selectively inhibit the phosphatases, protein kinases, or ectonucleotidases, which may contribute to their anti-cancer, anti-viral, and anti-bacterial activities. For instance, POMs have been found to inhibit nucleases as well as DNA- and RNA-polymerases in in vitro assays, and the inhibition effect on HIV has been extensively studied. Additionally, some POMs have been found to possess inhibitory effects on the pathogenic bacterium *Legionella pneumophila* at very low concentrations [11,12,13,14]. Recent studies have indicated that the anti-tumor mechanism may be related to bioactivities such as the induction of apoptosis, inhibition of related enzymes, and DNA binding effects.

Cancer is one of the major reasons for death, due to its rapid pace of development and the lack of efficient treatments. “China Cancer Statistics 2015” reported in the magazine *A Cancer Journal for Clinicians* that in 2015 there were 4.292 million and 2.814 million new cases of cancer and deaths of cancer in China, respectively. The most common types of cancer found in women are breast, lung, gastric, colorectal, and esophageal cancer [15]. Among all cancer types, breast cancer was the most common in female patients. Furthermore, breast cancer is the leading cause of death in women and fifth overall, so there is an urgent need to find efficient drugs to treat breast cancer.

Cancers are highly proliferative tissues, due to excessive gene amplification that leads to DNA damage and causes genetic mutations or changes in chromosome structure. DNA is a very important genetic substance in organisms and a primary intercellular target for anti-tumor drugs. Anti-cancer drugs combine with the DNA of cancer cells by electrostatic binding, groove binding, or intercalation to contribute to damage and breakage to the DNA double helix structure of cancer cells. For instance, Dianat and co-workers studied the calf thymus DNA (*ct*DNA) binding affinity and anti-tumor activities of three types of POMs by spectroscopic methods and 3-[4,5-dimethylthiazol-2-yl]-2,5-diphenyltetrazolium bromide (MTT) assays. The results showed that DNA binding affinity played an important role in the antitumor activity of POMs [16]. Furthermore, human serum albumin (HSA)—a large molecule which is the most abundant protein in plasma, and can function with many endogenous and exogenous substances—exerts physiological functions such as storage and trans-shipment. HSA is the most important drug transport carrier in plasma. Drugs can be reversibly combined with serum albumin and form drug–protein complexes, as a kind of temporary storage form of the drug in organisms that can effectively avoid the quick metabolism and elimination of drugs, in order to maintain the blood drug concentration and effective time. Bovine serum albumin (BSA) is one of the bovine serum globulins that has been widely used as a replacement for HSA in evaluating the interactions of drugs with proteins due to its medical importance, low cost, availability, ligand-binding properties, and wide acceptance in the pharmaceutical industry, the most important reason being that HSA and BSA have 76% sequence homology and homologous disulphide bond arrangements which make the structure and bioactivity of BSA similar to that of HSA [17,18]. Therefore, studies on the interactions of POMs with biological molecules have made important contribution to studying the structures and functions of bio-macromolecules and some biophysical processes.

As a part of our ongoing anti-tumor drug discovery program [19], a number of POMs have been synthesized and evaluated for their potential anti-tumor activities. Herein, a polyoxovanadate, K_12_[V_18_O_42_(H_2_O)]∙6H_2_O (**V_18_**), was prepared and characterized. The anti-tumor activities of **V_18_** against MCF-7 cells were investigated in vitro using MTT assays. The apoptosis of breast cancer cells was investigated by confocal laser scanning microscopy. The flow cytometry method was used to analyze the impact on the cell cycle. The results show that **V_18_** possesses strong anti-cancer activities against MCF-7 cells. The *ct*DNA, BSA, and HSA binding effects of **V_18_** were investigated using UV-Vis absorption spectroscopy and fluorescence quenching.

## 2. Results and Discussion

### 2.1. The Growth Inhibition Effect in Vitro Assays

**Figure 1:**
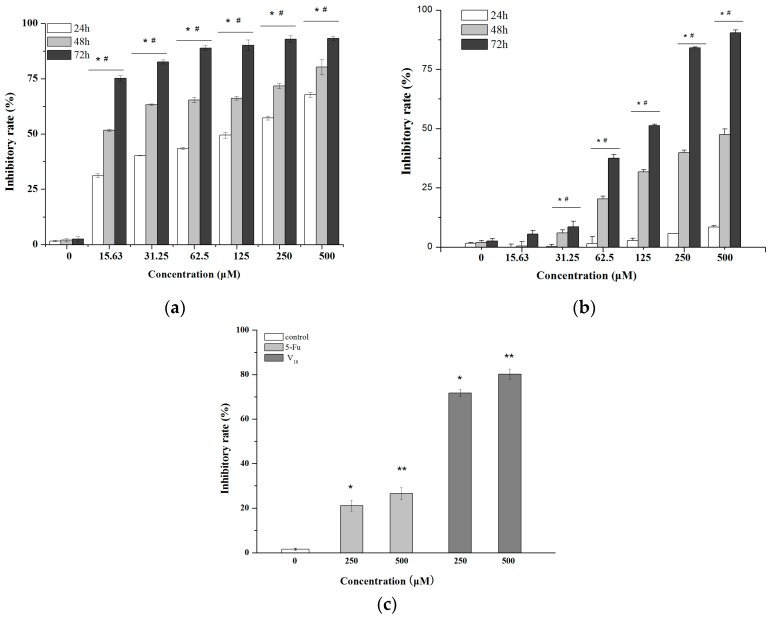
Inhibitory effect of **V_18_** with different concentrations on (**a**) MCF-7 cells and (**b**) MDA-MB-231 cells for 24 h, 48 h and 72 h. * *p* < 0.05 for **V_18_** at different time and doses compared to 0 μM group. # *p* < 0.05 for **V_18_** in the same dose at different times. (**c**) Inhibitory effects of **V_18_** and 5-fluorouracil (5-Fu) on MCF-7 cells with the concentration of 250, 500 μM at 48 h. * *p* < 0.05 for **V_18_** and 5-Fu at 250 μM compared to 0 μM group. ** *p* < 0.05 for **V_18_** and 5-Fu at 500 μM compared to 0 μM group.

Polyoxometalates containing different vanadium, K_5_PMo_10_V_2_O_40_, α-Na_10_[PW_11_V(H_2_O)O_37_]·16H_2_O, α-K_5_[SiW_11_VO_40_], α-1,2,3,-K_6_H[SiW_9_V_3_O_40_], K_12_[V_18_O_42_(H_2_O)]·6H_2_O, and α-Na_10_[PW_9_V_3_(H_2_O)O_37_]·16H_2_O, were screened for the anti-proliferation on MCF-7 based on the possible biological activities of vanadate. The values of IC_50_ at 24 h to MCF-7 cancer cells were 199.48, >1000, >1000, >1000, 45.95, 315.81 μM for K_5_PMo_10_V_2_O_40_, α-Na_10_[PW_11_V(H_2_O)O_37_]·16H_2_O, α-K_5_[SiW_11_VO_40_], α-1,2,3,-K_6_H[SiW_9_V_3_O_40_], K_12_[V_18_O_42_(H_2_O)]·6H_2_O, and α-Na_10_[PW_9_V_3_(H_2_O)O_37_]·16H_2_O, respectively. The results show that the **V_18_** exist a higher anti-proliferation effect on MCF-7 cell line. Therefore, the inhibitory rates in control group, **V_18_** groups, and 5-fluorouracil (5-Fu) groups were measured at different concentrations and times on MCF-7 and MDA-MB-231 cancer cell lines, respectively. As shown in Figure 1a,b, the MCF-7 and MDA-MB-231 cell viabilities decreased as the concentrations of **V_18_** were increased. The 50% inhibition concentrations (IC_50_) of **V_18_** against MCF-7 and MDA-MB-231 were 45.95 μM and >500 μM for 24 h, 11.95 μM and 360.32 μM for 48 h, and 12.49 μM and 135.66 μM for 72 h, respectively. Because the MTT results of **V_18_** on MCF-7 are better than on MDA-MB-231, further anti-proliferative studies were concentrated on MCF-7. As is shown in Figure 1c, the antitumor activity of **V_18_** was significantly stronger than that of 5-Fu for 48 h at the concentrations of 250, 500 μM (*p* < 0.05).

### 2.2. Morphological Analysis

**Figure 2:**
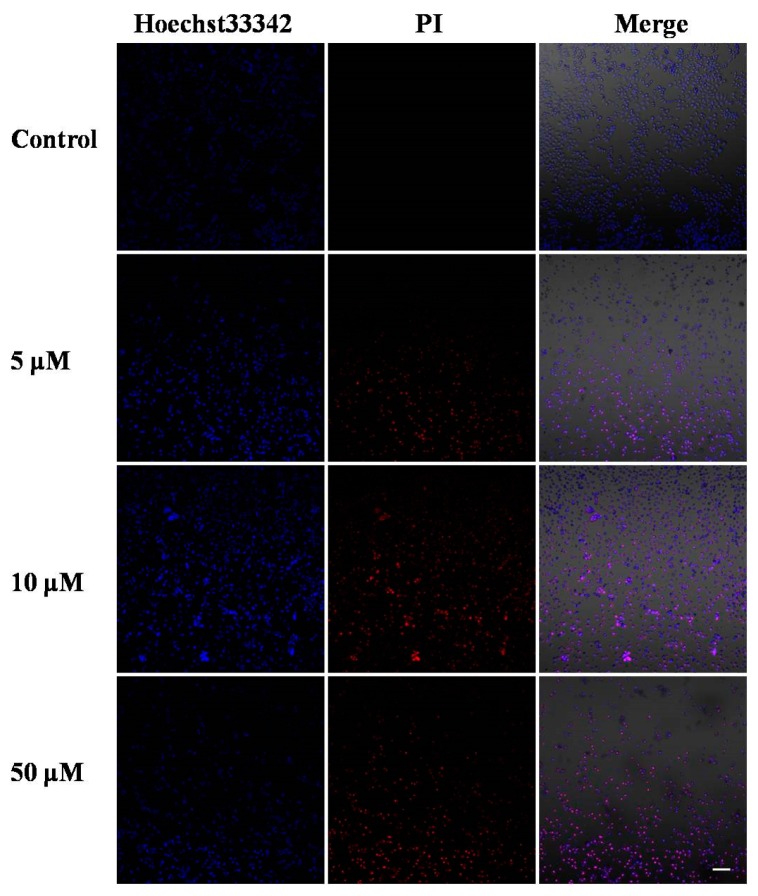
Morphological changes of MCF-7 cells by Hoechst33342 and PI staining treated with 0, 5, 10, and 50 μM of **V_18_**. Scale bar: 50 μM.

Hoechst33342 staining was performed to see the morphology changes in the MCF-7 cell nuclei, and whether **V_18_** could induce apoptosis in MCF-7 cells was investigated by dyeing with Hoechst33342/PI. (Propidium Iodide) In the Hoechst33342/PI double staining assay, the cells can be stained by Hoechst33342 to blue and the nucleus can be stained by PI to red. Therefore, the normal cells were light blue, the apoptotic cells were brilliant blue and light red, and dead cells were brilliant red. After the cell lines were treated with **V_18_** at 0 μM, 5 μM, 10 μM, and 50 μM doses, variable cells, apoptotic cells, and necrotic cells could be found. As the dose of **V_18_** was increasing, cell nucleus shrank, and increasing numbers of necrotic and apoptotic cells appeared. These results showed that compound **V_18_** clearly induced apoptosis and necrosis in MCF-7 cells in a dose-dependent manner (Figure 2).

### 2.3. Flow Cytometric Analysis for Cell Cycle Distribution and Apoptosis

**Figure 3:**
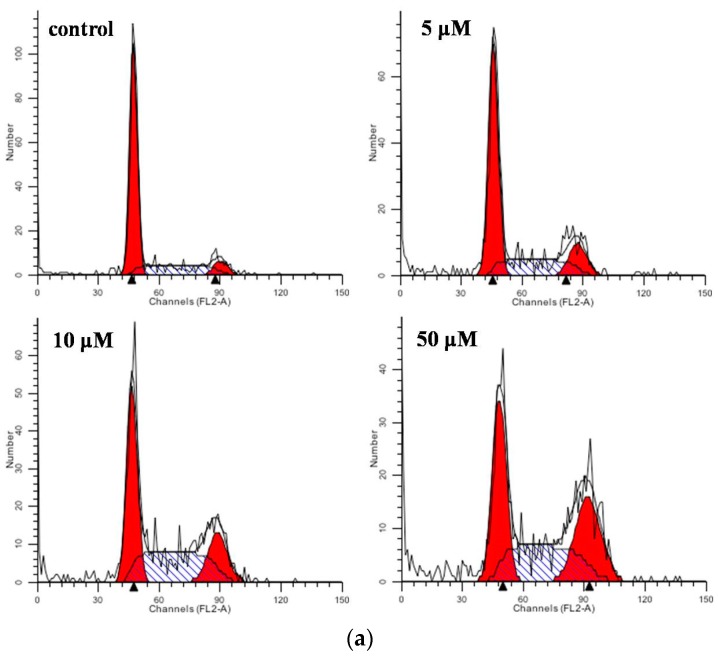

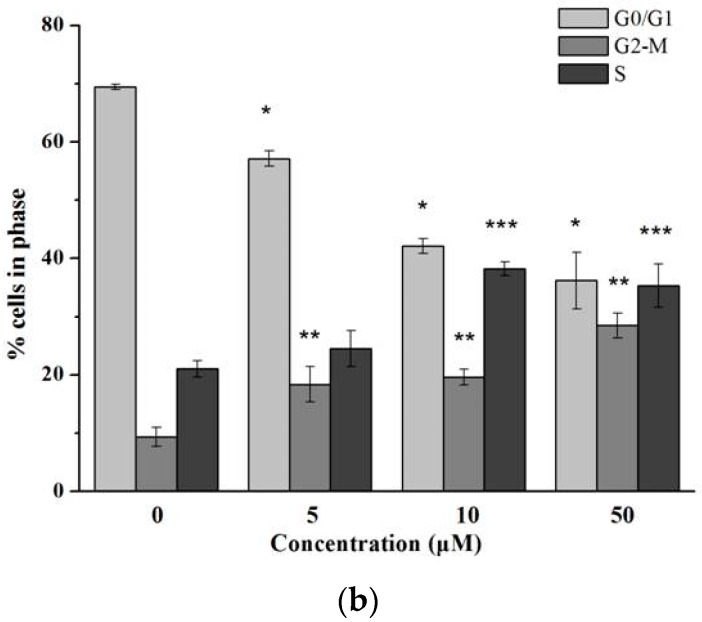
The cycle analysis of MCF-7 cell treated with **V_18_** for 24 h. Data are presented as the mean ± SD (*n* = 3). (**a**) The cycle analysis of MCF-7 cells treated with 0, 5, 10, 50 μM **V_18_**; (**b**) The cell rate of G_0_/G_1_, S, and G_2_-M phase cells treated with 0, 5, 10, 50 μM **V_18_**. * *p* < 0.05 for G_0_/G_1_ phase compared to control group. ** *p* < 0.05 for G_2_-M phase compared to control group. *** *p* < 0.05 for S phase compared to control group.

To further explore the mechanism for the inhibition effect on MCF-7 cells of **V_18_**, the experiments on changes of cell cycle and apoptosis were performed. 1 × 10^6^ MCF-7 cells were seeded in 12-well-plates and treated with **V_18_** in concentrations of 0, 5, 10 and 50 μM for 24 h and then analyzed by flow cytometry. The cell cycle results are shown in Figure 3; MCF-7 cells at the G_1_ phase were 69.44%, 57.13%, 42.12% and 36.19% for various concentrations of **V_18_**. MCF-7 cells at the G_2_/M phase were 9.35%, 18.35%, 19.62%, and 28.47% and cells at the S phase were 21.05%, 24.51%, 38.25%, and 35.33% for various concentrations of **V_18_**, respectively.

**Figure 4:**
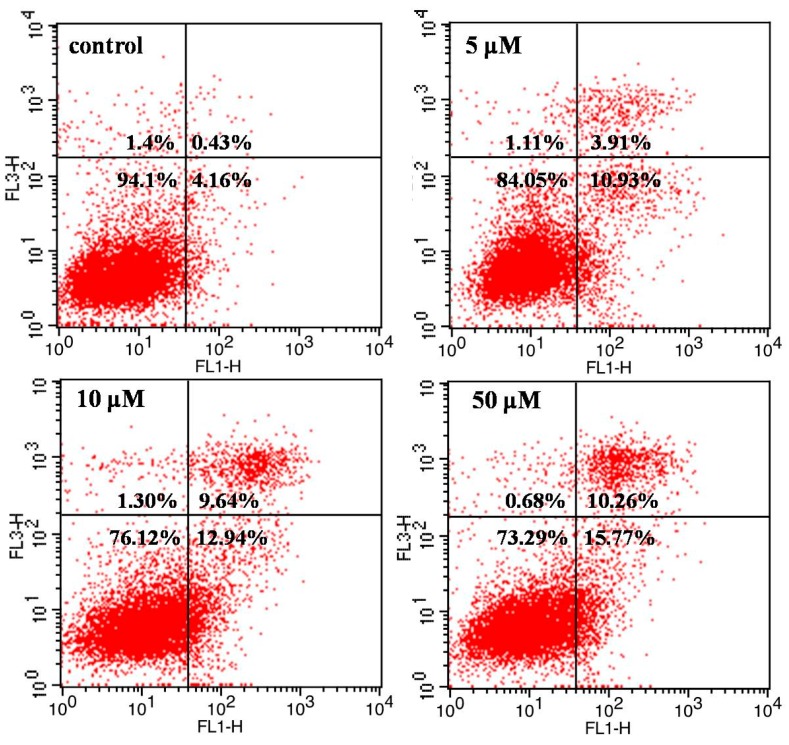
The apoptosis analysis of MCF-7 cell treated with **V_18_** for 24 h.

The results indicated that **V_18_** can alter the cell cycle, contribute to increasing the number of cells in G_2_/M phase and S phase, and also reduce the number of G_1_ phase cells. In comparison with control group, **V_18_** can induce G_2_/M phase cell cycle arrest in MCF-7 cells (*p* < 0.05). To further investigate the induction of apoptosis on MCF-7 cells by **V_18_**, the results are shown in Figure 4. In the flow cytometric analysis results, the upper left square shows cells with mechanical damage, the upper right shows late apoptotic and necrotic cells, the lower left shows normal live cells, and the lower right shows early apoptosis cells. It can be found that as the **V_18_** concentrations were increased the percentage of apoptotic MCF-7 cells increased to 15.77% and the late apoptotic and necrotic cells percentage increased to 10.26% compared to 4.16% and 0.43% in the control group, which was consistent with the results of morphological analysis. Those results indicated that **V_18_** can induce the apoptosis and necrosis which contribute to inhibiting breast cancer.

### 2.4. The Expression of Apoptosis-Related Molecules

**Figure 5:**
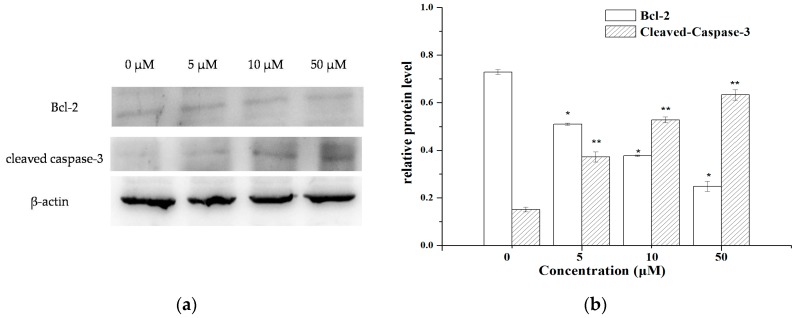
(**a**) The expression of apoptosis-related proteins Bcl-2, caspase-3, and cleaved caspase-3 in MCF-7 cells treated with 0, 5, 10, 50 μM **V_18_**; (**b**) The protein content normalized by β-actin. Apoptosis-related proteins were detected with Western blotting utilizing specific antibodies. * *p* < 0.05 compared to Bcl-2 of the control group; ** *p* < 0.05, compared to cleaved caspase-3 of the control group.

The flow cytometry analysis results suggested that the apoptosis pathway might be an important mechanism for the inhibitory effect of **V_18_** against MCF-7 cells. Therefore, Western blot assays were performed to test the expression level of the apoptosis-related proteins Bcl-2 and cleaved-caspase-3 to further explore the mechanism. As shown in Figure 5, the results showed that the expression of Bcl-2 was decreased and the cleaved caspase-3 was increased with the increasing **V_18_** concentrations, which was consistent with the changes in the rate of cell apoptosis. These results suggested that **V_18_** might induce MCF-7 cell apoptosis by up-regulating caspase-3 and down-regulating Bcl-2.

### 2.5. ctDNA Binding of **V_18_**

**Figure 6:**
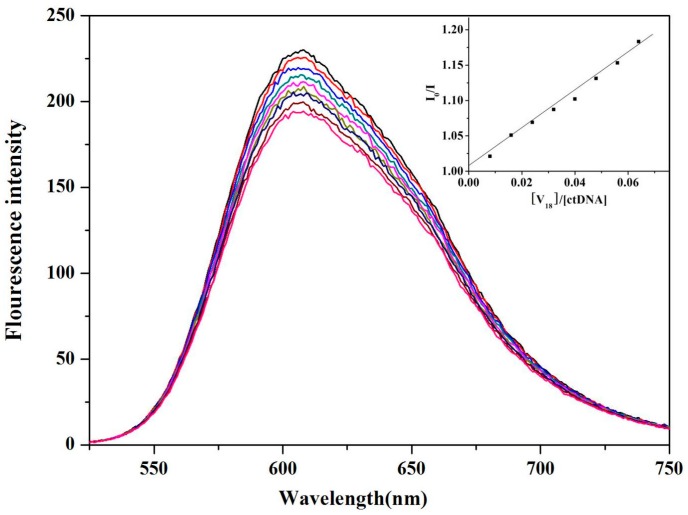
The fluorescence spectra of mixed EB (20 μM) and calf thymus DNA *(ct*DNA; 100 μM) in the presence of different concentrations of **V_18_** (0, 1.33, 2.67, 4.00, 5.33, 6.67, 9.33, 10.67, 12 μM) in phosphate-buffered saline (PBS). It shows the fluorescence intensity changes with increasing concentrations of **V_18_**. Insert: Stern–Volmer plot of the fluorescence titration data of EB-*ct*DNA with different concentrations of **V_18_** in PBS.

The binding characteristics of **V_18_** with *ct*DNA were investigated by the fluorescence titration method. EB (Ethidium bromide) was used as a fluorescence probe, and the fluorescence emission of EB bound to *ct*DNA was detected in the presence of different concentrations of **V_18_**. The results in Figure 6 show the quenching effect of **V_18_**. Plots of F_0_/F versus [**V_18_**] can be divided into a straight line (the inset of Figure 6) and a value of 2.85 × 10^4^ (*R*^2^ = 0.9956) was obtained for Stern–Volmer quenching constants. The binding constant of **V_18_** to *ct*DNA is lower than that of the classical probe EB. This result indicates that the binding mode was not an intercalation interaction, but rather groove or outside stacking binding.

### 2.6. BSA and HSA Binding of **V_18_**

**Figure 7:**
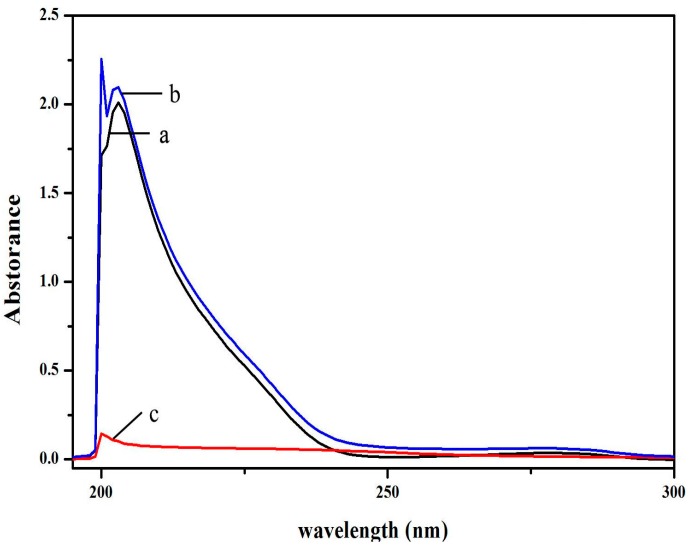
The effect between **V_18_** and bovine serum albumin (BSA). UV-Vis spectra of (a) 1.25 μM BSA, (b) **V_18_**-BSA with molar ratio of 1:1, and (c) 1.25 μM **V_18_**.

The results of BSA binding absorption of **V_18_** are shown in Figure 7. The spectra of 1.25 μM **V_18_**, free BSA, and 1:1 **V_18_**-BSA solutions were all recorded. The characteristic absorption peak of BSA was near 208 nm and **V_18_** at 202 nm. The results showed that the absorption peak of BSA was slightly increased due to the addition of **V_18_**. This indicated that there might exist an interaction between **V_18_** and BSA.

**Figure 8:**
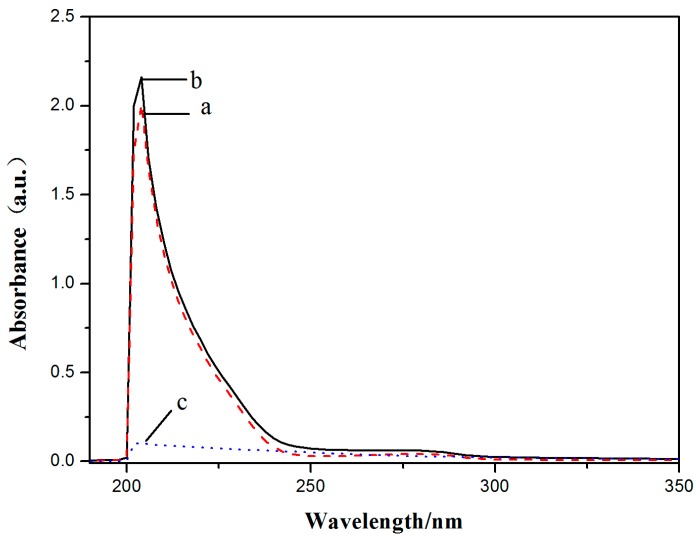
The effect between **V_18_** and human serum albumin (HSA). UV-Vis spectra of (a) 0.5 μM HSA, (b) **V_18_**-HSA with molar ratio of 1:1, and (c) 0.5 μM **V_18_**.

The spectra of 0.5 μM **V_18_**, free HSA, and 1:1 **V_18_**-HSA solutions are shown in Figure 8. The characteristic absorption peak of HSA was near 210 nm, and results showed that the absorption peak of HSA was slightly increased because of the **V_18_**. This indicated that there might exist an interaction between **V_18_** and BSA and a complex might be formed.

**Figure 9:**
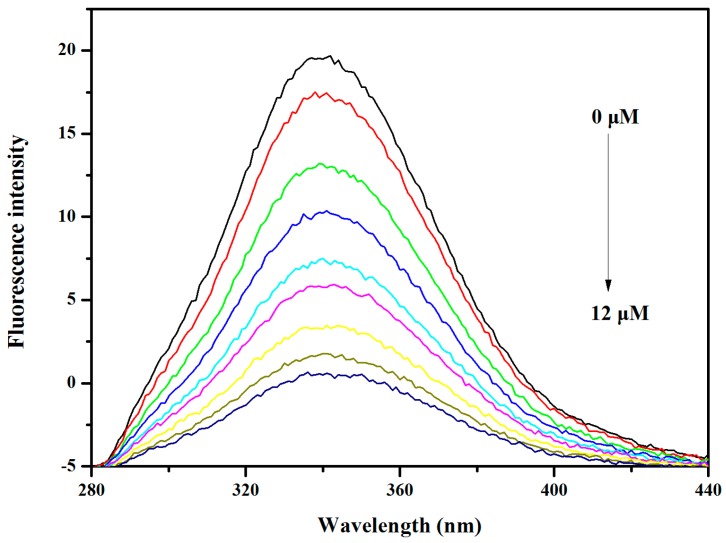
The fluorescence spectra of BSA (0.5 μM) in the presence of different concentrations of **V_18_** (0, 1.33, 2.67, 4.00, 5.33, 6.67, 9.33, 10.67, 12 μM) in PBS.

The fluorescence spectra showed a peak of BSA at about 340 nm. The fluorescence results of BSA and **V_18_** are given in Figure 9.

**Figure 10:**
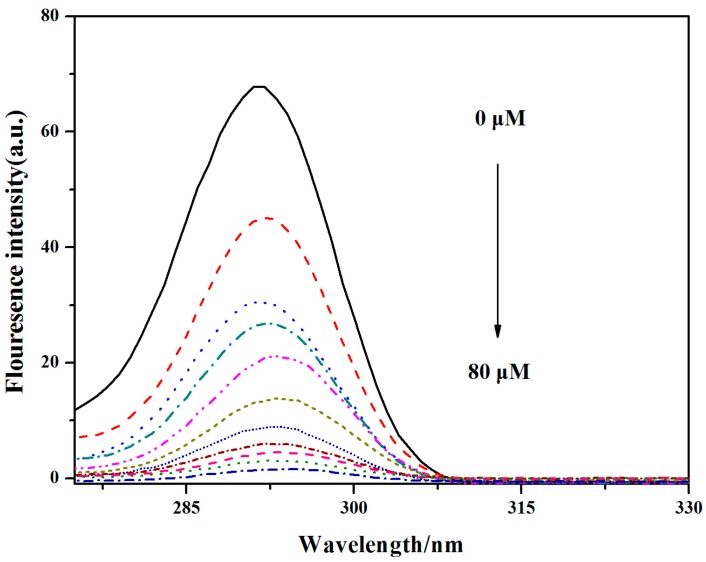
The fluorescence spectra of HSA (2 μM) in the presence of different concentrations of **V_18_** (0, 8, 16, 24, 32, 40, 48, 56, 64, 72, 80 μM) in PBS.

According to the **V_18_** increase, the fluorescence intensity of BSA was sequentially decreased. The peak of BSA was slightly red shifted and decreased, which showed that the microenvironment around BSA was changed due to the **V_18_**. There are two kinds of fluorescence quenching, including dynamic and static quenching [20,21]. Due to the results, the absorbance peak of BSA was reduced by **V_18_**, and the fluorescence quenching of **V_18_** to BSA was likely to be static quenching. The fluorescence spectra results in Figure 10 show an HSA peak at about 292 nm. As the **V_18_** concentration increased, the fluorescence intensity of HSA progressively decreased, like in BSA. The peak of HSA was slightly red shifted and decreased, which indicated that the microenvironment around HSA was changed due to the **V_18_** and the fluorescence quenching of **V_18_** to HSA was likely to be due to static quenching. 

Synchronous fluorescence (SFS) experiments were performed to study the binding effect between **V_18_** and BSA. Synchronous fluorescence can provide information about the tyrosine and tryptophan residues of BSA when the excitation wavelength and emission wavelength interval was established at 15 and 60 nm, respectively.

**Figure 11:**
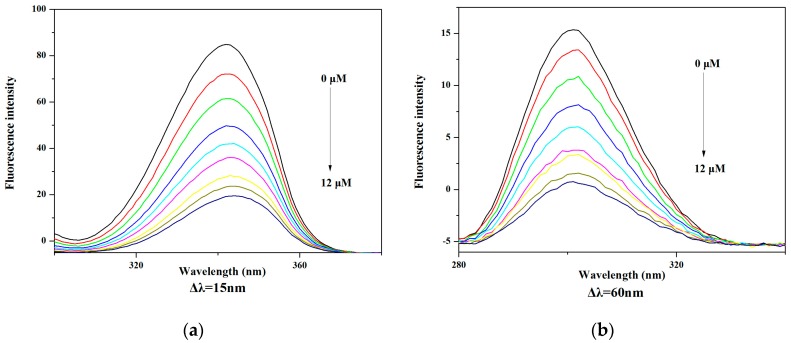
The synchronous fluorescence spectra of BSA (0.5 μM) in the presence of different concentrations of **V_18_** (0, 1.33, 2.67, 4.00, 5.33, 6.67, 9.33 10.67, 12 μM): (**a**) Δλ = 15 nm and (**b**) Δλ = 60 nm at 20 °C in PBS.

In Figure 11a (Δλ = 15 nm) and Figure 11b (Δλ = 60 nm), the emission peaks of tryptophan residues can be observed slightly red shifted. The results suggested that the conformation of the BSA was altered. The hydrophobicity of tryptophan and tyrosine residues decreased in the microenvironment [22,23]. The results show that the tyrosine and tryptophan fluorescence intensity fell and was slightly red shifted at the same time, the tyrosine residues in **V_18_**-BSA solution decreased more obviously, suggesting that the binding site between **V_18_** and BSA was closer to tyrosine residues than tryptophan.

**Figure 12:**
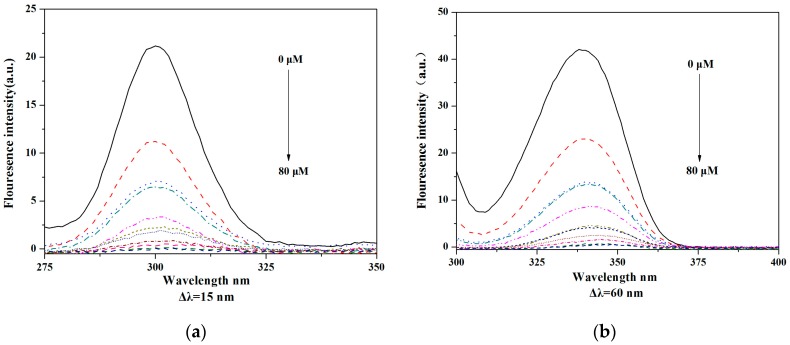
The synchronous fluorescence spectra of HSA (2 μM) in the presence of different concentrations of **V_18_** (0, 8, 16, 24, 32, 40, 48, 56, 64, 72, 80 μM): (**a**) Δλ = 15 nm and (**b**) Δλ = 60 nm at 20 °C in PBS.

As shown in Figure 12, tyrosine and tryptophan fluorescence intensity fell and were slightly red shifted at the same time, and the tyrosine residues in **V_18_**-HSA solution decreased more obviously, suggesting the binding site between **V_18_** and HSA was closer to tyrosine residues than tryptophan ones. Those results indicated that the conformation of the HSA was altered. The hydrophobicity of tryptophan and tyrosine residues decreased in the microenvironment [22,23].

## 3. Materials and Methods

### 3.1. Chemicals and Reagents

All solvents and chemicals used in the experiment were obtained from commercial suppliers and used as purchased without further purification. Penicillin–streptomycin (Sigma, Shanghai, China), DMEM (Hyclone, Logan, UT, USA), fetal bovine serum (Clark, Greensboro, NC, USA), 3-[4,5-dimethylthiazol-2-yl]-2,5-diphenyltetrazolium bromide (MTT, Sigma), DMSO (Sigma), and phosphate buffered saline (Sigma) were used for the determination of POM cytotoxicity. The PI and Hoechst33342 dye were purchased from Dingguo Chemicals Company (Chanchun, China) and Beyotime Chemicals Company (Shanghai, China). The *ct*DNA and EB were purchased from Sigma. The solution of *ct*DNA was prepared by dissolving *ct*DNA in phosphate buffer solution at 4 °C under intense stirring for more than 24 h to get a homogeneous solution. Solutions of *ct*DNA in 10 mM phosphate buffer solution (PBS, 10 mM) of pH 7.4 gave a ratio of UV absorbance at 260 nm and 280 nm, and A_260_/A_280_ was 1.88, indicating that the *ct*DNA was sufficiently free of protein and needed no further purification. The *ct*DNA concentration per nucleotide was determined by UV absorbance at 260 nm (ε_260_ = 6600 L·mol^‒1^·cm^‒1^). The HSA was purchased from Sigma.

### 3.2. Synthesis of K_12_[V_18_O_42_(H_2_O)]∙6H_2_O

The polyoxovanadate K_12_[V_18_O_42_(H_2_O)]∙6H_2_O (**V_18_**) was prepared according to the literature [24], and was identified by IR spectroscopy (Nicolet Magna 560 IR spectrometer, Nicolet, Madison, WI, USA) and elemental analysis. The characteristic IR peaks of the **V_18_** (KBr pellet, cm^−1^) were 3443, 2352, 2014, 1612, 1270, 979, 906, 782, 666, and 520.

### 3.3. Cell Culture and the Antitumor Activity in Vitro Assay

MCF-7 and MDA-MB-231 breast cancer cells were maintained in DMEM culture medium with 10% FBS by monolayer in a 5% CO_2_ humidified atmosphere at 37 °C. The antitumor activity of **V_18_** on the breast cancer cells was analyzed by the MTT assay [23,24]. MCF-7 and MDA-MB-231 cells were seeded in 96-well plates of 5 × 10^5^ cells/mL. The cells were then exposed to a series of concentrations of **V_18_** for 24, 48 or 72 h under conditions of 37 °C, 5% CO_2_, after which 20 μL of a 5 mg/mL MTT solution was added for 4 h. Then, 150 μL of DMSO was added to every well to dissolve the blue crystals adequately. The effects of the clinical anti-cancer drug 5-fluorouracil (5-Fu) on the cell inhibition of MCF-7 cells at 48 h was also determined using the same method. The absorbance was detected at 490 nm on a microplate reader (Biotek Co, Winooski, VT, USA). The cytotoxicity was measured by the reduction of MTT observed in mitochondria at 24, 48 and 72 h after the initial treatment.

### 3.4. Morphological Observation

MCF-7 cells were grown in 24-well confocal culture dishes and treated with three different concentrations (50, 10 and 5 μM) of **V_18_** for 24 h at 37 °C, 5% CO_2_. After that, the apoptotic cells were detected by staining with Hoechst 33342 and PI [25]. The cells were observed by fluorescence confocal microscopy (Olympus FV1000, Tokyo, Japan).

### 3.5. Flow Cytometric Analysis of Cell Cycle Distribution and Apoptosis

MCF-7 cells were seeded in 12-well plates at a density of 1 × 10^6^ cells per well. After 24 h, the cells were treated with **V_18_** at the concentrations of 5, 10 and 50 μM for 24 h. The percentage of cells in G_1_, S and G_2_/M phases and the apoptosis were calculated using a flow cytometer [26] (Epics XL ADC, Beckman Coulter, Miami, FL, USA).

### 3.6. Western Blot Analysis

MCF-7 cells were seeded in 6-well plates at a density 2 × 10^6^ cells per well. After 24 h, different doses of **V_18_** were added to each well for 24 h. Then, the total protein was extracted and the protein concentrations were established using the BCA assay kit. SDS-PAGE was used to separate the protein and then transfer it to a PVDF membrane. The membranes were incubated with primary antibodies, caspase-5, Bcl-2, and β-actin, and then incubated with the secondary antibodies. Bands were visualized using enhanced chemiluminescence detection reagents, and scanned images were quantified using the ImageJ software.

### 3.7. ctDNA Binding Experiments

The absorption titration spectra were recorded on a U3010 UV-Vis spectrophotometer (Hitachi, Tokyo, Japan) by sequential addition of a specified volume of *ct*DNA stock solution into a 1 cm path length cuvette containing **V_18_** solution (10 μM). After every addition of *ct*DNA solution, the absorption spectra were recorded from 190 to 600 nm. The intrinsic binding constant of compounds with *ct*DNA was determined by the equation from [27,28]. 

All fluorescence spectra were recorded on a fluorescence spectrophotometer (Shimadzu, Tokyo, Japan) using a 1 cm quartz cuvette in the wavelength range from 190 to 600 nm, and 522 nm was chosen as the excitation wavelength. Excitation and emission slit widths were set as 5 and 10 nm, respectively. The experimental data were plotted according to the Stern–Volmer equation in [29,30]. In competition binding experiments, the concentrations of EB and *ct*DNA were 20 μM and 100 μM, respectively, while the **V_18_** concentration was varied from 0 to 12 μM. 

### 3.8. BSA and HSA Binding Experiments

The UV absorption spectra were recorded on a UV-Vis spectrophotometer using 1 cm quartz cuvettes in the wavelength range from 190 to 600 nm. The concentrations of **V_18_** and BSA were both 1.25 μM, and the mixture of **V_18_** and BSA had a molar ratio of 1:1. A 0.5 μM BSA solution was added in the sample pool, and the fluorescence spectra of BSA were recorded from 310 to 600 nm. Then, the same amount of **V_18_** was added into the sample pool step-by-step until the final concentration was 1.2 × 10^−5^ mol/L. The fluorescence spectra of different concentrations of **V_18_**-BSA were detected at 310–440 nm. At the same time, synchronous fluorescence spectra ware detected (Δλ values of 15 nm and 60 nm) and the excitation and emission were set as 5.0 and 5.0 nm.

For the HSA, the concentration of **V_18_** and HSA were both 0.5 μM, and the mixture of **V_18_** and HSA had a molar ratio of 1:1. The fluorescence spectra of HSA were recorded from 310 to 600 nm, and 2 μM HSA solution was added in the sample pool. Then, the same amount of **V_18_** buffer was added into the sample pool in sequence until the final concentration was 8 × 10^−5^ mol/L. The fluorescence spectra of different concentrations of **V_18_**-HSA were detected at 290–440 nm. At the same time, synchronous fluorescence spectra were detected.

### 3.9. Statistical Analysis

Data were expressed as mean ± SD. All experiments were performed in triplicate, unless otherwise indicated. Statistical significance was evaluated by one-way analysis of variance (ANOVA) combined with Duncan’s multiple range tests. The IC_50_ values represent the means of quadruplicate determination ± standard deviation (SD). MTT analysis of multiple comparisons were statistically analyzed (*p* < 0.05) using the SPSS 13.0 software (SPSS Inc., Chicago, IL, USA.).

## 4. Conclusions

In summary, the antitumor activities of the polyoxovanadate K_12_[V_18_O_42_(H_2_O)]∙6H_2_O (**V_18_**) were investigated. The inhibitory effect of **V_18_** on MCF-7 cells was higher than that of 5-Fu (*p* < 0.05), and exhibited a dose-dependent trend. The anti-tumor activity of **V_18_** was attributable to the DNA binding effect, BSA binding characteristic, HSA binding effect, induction of apoptosis, and cell cycle G_2_/M phase arrest (*p* < 0.05). Additionally, the DNA binding effects, BSA binding characteristics, and HSA binding effects might enhance the inhibitory effect of **V_18_** against MCF-7 cells because **V_18_** can bind to *ct*DNA by groove or outside stacking binding modes and change the microenvironment around BSA and HSA.
